# New Route to Longer Life

**DOI:** 10.1371/journal.pbio.0020308

**Published:** 2004-08-24

Ever since the early Greeks recast humans as the center of the universe and remade God in their own image, Western philosophers and poets have grappled with the limits of human mortality. Philosophers found relief from Keats's “unwilling sleep” by dividing human existence into body and soul and asserting that the true essence of humanity lies in the immortal soul, not in the body. Ironically, as this decidedly nonscientific subject has lost favor with modern-day philosophers, it has captured the imagination of scientists. But, for now at least, the interest is in prolonging life rather than escaping mortality.

Over the past twenty years, mounting evidence from a wide range of organisms indicates that a longer life awaits those who eat less. In yeast, calories can be restricted directly, by limiting yeast's glucose supply, or indirectly, by inhibiting yeast's ability to metabolize glucose. Either way, many studies have suggested that the increased longevity associated with calorie restriction is linked to increased activity of a gene called *SIR2*. Now, Brian Kennedy and colleagues show that calorie restriction and *SIR2* promote longevity through distinct genetic pathways—and that aging in yeast and higher organisms may be more similar than previously thought.

One of the causes of aging in yeast is the accumulation of coiled bits of DNA, called extrachromosomal ribosomal DNA circles (ERCs), in the nucleus of a mother cell (which divides to create two identical daughter cells). An overabundance of these rDNA circles wreaks havoc on a cell and eventually kills it. Genetic mutations that reduce their levels are linked to increased life span. Mutations that disrupt the *FOB1* gene, for example, dramatically reduce ERC levels and increase the reproductive life span of cells by 30%–40%. In contrast, mutations that disrupt *SIR2* increase ERC levels and cut life span in half, while increasing *SIR2* activity increases life span by 30%–40%.

In previous experiments, several groups have identified a link between calorie restriction, *SIR2*, and the accumulation of ERCs. The idea is that calorie restriction somehow activates the protein encoded by *SIR2*, which in turn decreases ERC accumulation. Now, Kennedy's team has found that the combination of calorie restriction and *FOB1* mutation increases life span more than either approach does alone. This finding was unexpected because previous studies showed that combining increased *SIR2* activity with *FOB1* deletion mutations did not extend life span. If calorie restriction extends life through *SIR2*, then combining either caloric restriction or *SIR2* overexpression with *FOB1* mutations should produce the same result.

This contradiction raised the possibility that calorie restriction operates through another mechanism, independent of *SIR2*. In support of this view, caloric restriction enhances life span to a greater extent in *FOB1* mutants lacking *SIR2* than in *FOB1* mutants with an intact *SIR2* gene. This and other genetic experiments indicate that calorie restriction does not always work through *SIR2*.

That suggests, the authors explain, that calorie restriction functions either by regulating ERC levels or by some still unknown molecular pathway. They conclude that the enhanced longevity seen in calorie-restricted *FOB1* mutants is not related to ERCs, because these yeast strains already have low ERC levels. Since calorie restriction is the only demonstrated approach to increasing life span in a diverse range of organisms, including mammals, and since there's no evidence that ERCs affect the aging of any organism besides yeast, these results bode well for understanding how calorie restriction works in higher organisms. And the finding that calorie restriction and *SIR2* operate through genetically distinct pathways in yeast, the authors conclude, suggests that certain aspects of both pathways might have been conserved through evolution. Working out the details of these pathways in yeast is the first step toward understanding which, if any, of these components might enhance longevity in humans. Of course, as any student of Greek mythology knows, longevity without eternal youth comes with a price.

